# Autophagy Declines with Premature Skin Aging resulting in Dynamic Alterations in Skin Pigmentation and Epidermal Differentiation

**DOI:** 10.3390/ijms21165708

**Published:** 2020-08-09

**Authors:** Daiki Murase, Ayumi Kusaka-Kikushima, Akira Hachiya, Rachel Fullenkamp, Anita Stepp, Asuka Imai, Mizuki Ueno, Keigo Kawabata, Yoshito Takahashi, Tadashi Hase, Atsushi Ohuchi, Shuhei Nakamura, Tamotsu Yoshimori

**Affiliations:** 1 Biological Science Research, Kao Corporation, Haga 321-3497, Japan; ohuchi.atsushi@kao.com; 2 Biological Science Research, Kao Corporation, Odawara 250-0002, Japan; kusaka.ayumi@kao.com (A.K.-K.); ueno.mizuki@kao.com (M.U.); kawabata.keigo@kao.com (K.K.); takahashi.yoshito@kao.com (Y.T.); 3 Planning and Implementation, Kao Corporation, Haga 321-3497, Japan; akira.hachiya@kao.com; 4 Americas Research Laboratories, Kao USA Inc., Cincinnati, OH 45214, USA; rachel.fullenkamp@kao.com (R.F.); anita.stepp@kao.com (A.S.); asuka.imai@kao.com (A.I.); 5 Core Technology Sector, Kao Corporation, Sumida 131-0044, Japan; hase.tadashi@kao.com; 6 Department of Genetics, Graduate School of Medicine, Osaka University, Osaka 565-0871, Japan; shuhei.nakamura@fbs.osaka-u.ac.jp (S.N.); tamyoshi@fbs.osaka-u.ac.jp (T.Y.); 7 Department of Intracellular Membrane Dynamics, Graduate School of Frontier Biosciences, Osaka University, Osaka 565-0871, Japan

**Keywords:** autophagy, melanosome, keratinocyte, skin, hyperpigmentation, aging

## Abstract

Autophagy is a membrane traffic system that provides sustainable degradation of cellular components for homeostasis, and is thus considered to promote health and longevity, though its activity declines with aging. The present findings show deterioration of autophagy in association with premature skin aging. Autophagy flux was successfully determined in skin tissues, which demonstrated significantly decreased autophagy in hyperpigmented skin such as that seen in senile lentigo. Furthermore, an exacerbated decline in autophagy was confirmed in xerotic hyperpigmentation areas, accompanied by severe dehydration and a barrier defect, which showed correlations with skin physiological conditions. The enhancement of autophagy in skin ex vivo ameliorated skin integrity, including pigmentation and epidermal differentiation. The present results indicate that the restoration of autophagy can contribute to improving premature skin aging by various intrinsic and extrinsic factors via the normalization of protein homeostasis.

## 1. Introduction

Autophagy (literally “self-eating”) is a natural membrane traffic system related to the degradation of unnecessary cellular components, occurring in all cells in the body, and is known to have an essential role in providing fresh cellular components through protein recycling as well as other diverse functions [1,2]. However, autophagy activity has been found to be decreased in aged subjects [3], thus attempts to increase it in older individuals are considered warranted for suppression of the effects of aging and age-related disease.

Skin, the largest organ in the body, is not exempt from the effects of aging. In addition to intrinsic aging, skin is highly susceptible to tissue damage, as represented by pigment spots, wrinkles and sagging in elderly individuals, due to its continuous exposure to external stimuli. Most of those effects are accelerated by environmental factors, such as ultraviolet (UV) light, with the result known as photoaging. Moreover, transient but repeatable skin damage can accumulate and cause chronic changes in skin, which are characterized by various phenotypic conditions including xerosis, eczema and post-inflammatory pigmentation, as well as others.

With regard to the role of autophagy in skin homeostasis, it seems to target different types of organelles and proteostasis pathways in skin cells, such as nucleus elimination in differentiating keratinocytes [4], mitophagy in keratinocyte differentiation [5], anti-inflammation [6], and melanogenesis and antioxidative mechanisms in melanocytes [7,8,9]. However, the precise functions related to autophagy in skin have yet to be revealed.

Autophagy not only degrades endogenous substrates, but also serves as a response to incorporated exogenous targets in a physiological manner [10]. We recently reported significant role of autophagy-driven melanosome degradation in keratinocytes [11]. Melanosomes are a type of lysosome-related organelle (LRO) that store melanin, the most essential skin color determinant [12]. They are produced in epidermal melanocytes, followed by their distribution to neighboring keratinocytes. Although their amount in keratinocytes has a predominant impact on skin color, little is known about the detailed process that occurs following transfer to keratinocytes. In our study, melanosomes were shown to be subsequently degraded by canonical autophagy. Furthermore, it was noted that autophagic activity is associated with the extent of skin pigmentation, with lighter skin keratinocytes more capable of degrading melanosomes as compared with darker skin keratinocytes. Therefore, autophagy-targeted melanosome degradation is thought to not only be a highly conserved mechanism, regardless of skin color intensity, but also capable of generating variations in skin pigmentation, possibly dependent on its homeostatic balance in each context, though the detailed mechanisms require further investigation.

Based on previously presented findings, autophagy is considered to be essential for the homeostasis of proteins and organelles in skin. Nevertheless, an important question is how autophagy physiologically contributes to skin conditions in the context of skin aging and damage. In the present study, we investigated a potential role of autophagy by examining its activity in human skin tissues derived from a variety of premature skin aging phenotypes. The results revealed that autophagy declines with photoaging, such as hyperpigmentation, and was also found to be more deteriorated in ashy skin discoloration associated with severe skin xerosis. Furthermore, restoration of autophagy ameliorated melanosome degradation and epidermal differentiation ex vivo. These results indicate that autophagy plays a pivotal role in maintaining the integrity of human skin color by degrading melanosomes, which may occur as part of the dynamic homeostatic process of keratinocyte differentiation.

## 2. Results

### 2.1. Impairment of Autophagy in Senile Lentigo, a Common Type of Hyperpigmentation in Photoaging

**Figure 1:**
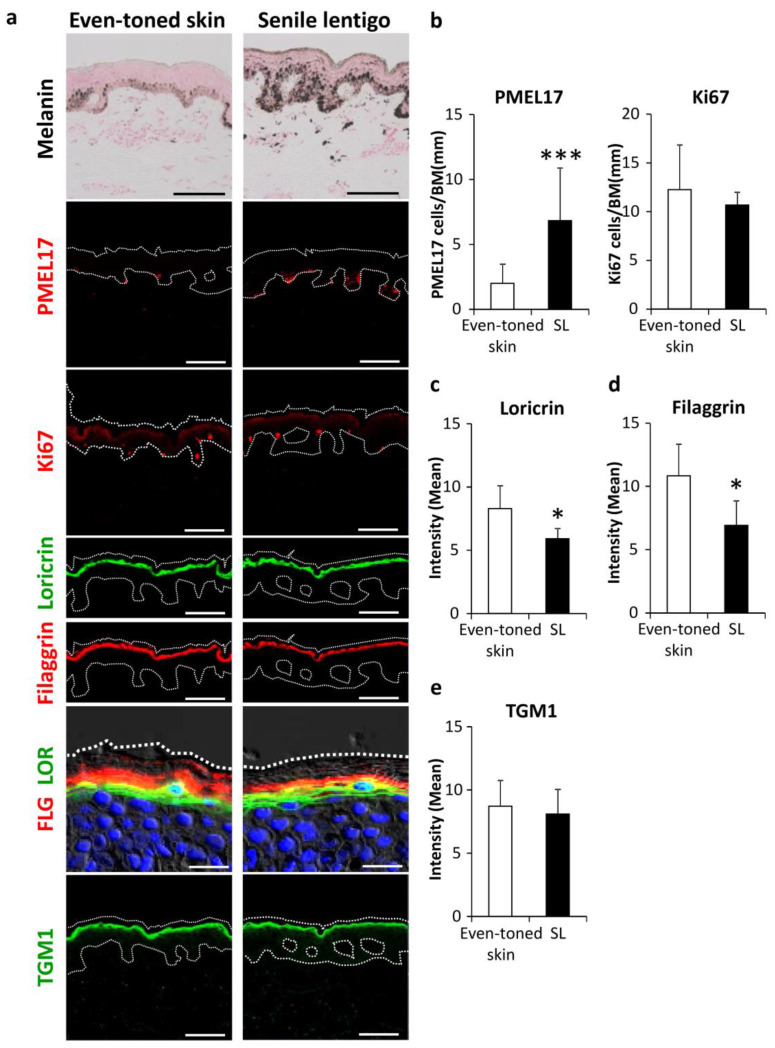
Enhancement of melanogenesis along with aberrant epidermal differentiation in senile lentigo regions. (**a**) Skin tissues were obtained from even-toned skin and senile lentigo regions of Japanese males (*n* = 8), and subjected to Fontana-Masson staining and immunofluorescent analysis. Merged images of PMEL17 (red), Ki67 (red), loricrin (green) and filaggrin (red), and magnified sets of filaggrin (FLG) (red) and loricrin (LOR) (green), transglutaminase 1 (TGM1) (green), and DAPI nuclear staining (blue) are shown. Bars = 100 μm or 20 μm (magnified for FLG/LOR). (**b**) Quantitative analysis of immunofluorescence of PMEL17-positve (left) and Ki67-positive (right) cells along the length of the basement membrane (BM). Values are shown as the mean ± SD (*n* = 8) *** *p* < 0.001 (paired t-test). Image analysis of immunofluorescence of loricrin (**c**), filaggrin (**d**) and transglutaminase 1 (TGM1) (**e**). Values are shown as the mean ± SD (*n* = 8) * *p* < 0.05 (paired t-test).

We investigated the involvement of autophagic deficiency in hyperpigmentation. Skin tissues were procured by biopsy from both senile lentigo (SL) and even-toned regions in each subject. Consistent with previous observations [13,14], histological analysis showed increased deposition of melanin pigmentation along with melanocyte proliferation in PMEL17-positive areas in the epidermis from SL regions (Figure 1a,b). It was also noted that the amounts of filaggrin and loricrin, both late epidermal differentiation marker proteins, were significantly reduced in SL as compared to even-toned regions of skin (Figure 1a,c,d). Transglutaminase 1 (TGM1), which is expressed earlier than other later stage markers, appeared to be similar between the tissue types (Figure 1a,e). These findings support similar previous observations, indicating that hyperpigmentation is likely accompanied by aberrant epidermal differentiation [15,16].

**Figure 2:**
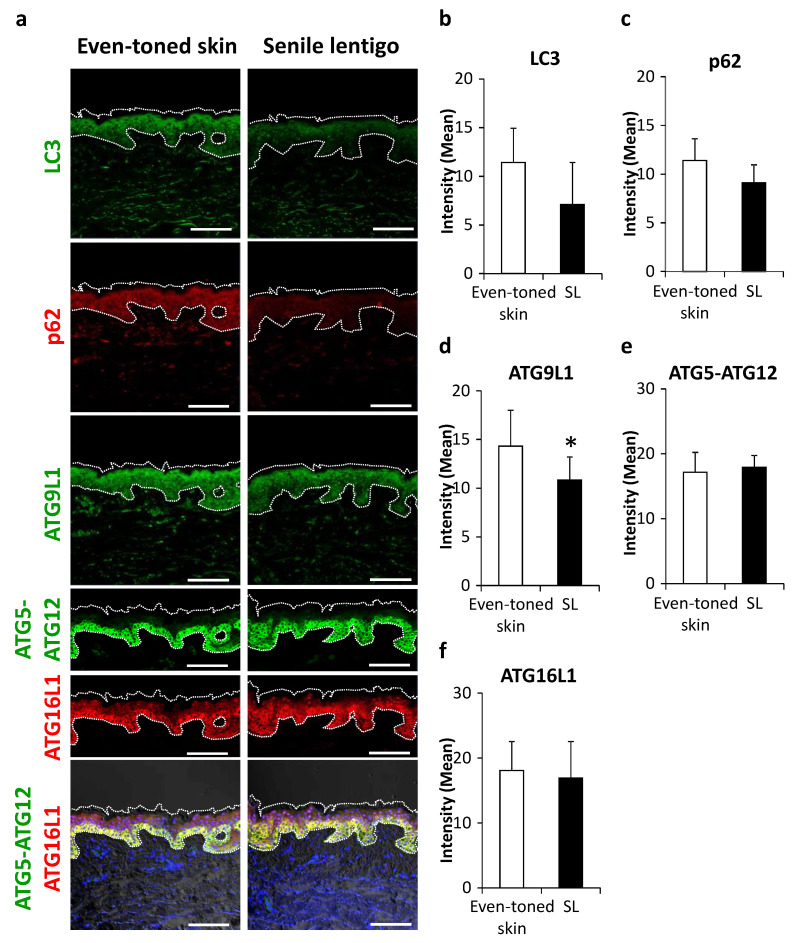
Changes in autophagy proteins in senile lentigo. (**a**) Skin tissues were obtained from even-toned skin and senile lentigo regions of Japanese males (*n* = 8), and subjected to immunofluorescent analysis. Merged image of LC3 (green), p62 (red), ATG9L1 (green), ATG5-ATG12 (green), ATG16L1 (red) and an overlaid set of ATG5-ATG12 (green), ATG16L1 (red), and DAPI nuclear staining (blue) are shown. Bars = 100 μm. Quantitative analysis of immunofluorescence of LC3 (**b**), p62 (**c**), ATG9L1 (**d**), ATG5-ATG12 (**e**) and ATG16L1 (**f**). Values are shown as the mean ± SD (*n* = 8) * *p* < 0.05 (paired t-test).

Subsequently, some essential proteins in autophagy machinery were visualized. LC3, an autophagosome marker, tended to be decreased in SL as compared with the even-toned skin regions (Figure 2a,b), with a similar trend seen for p62 protein, a substrate of autophagy (Figure 2a,c). Among a series of autophagy-related proteins (ATGs), ATG9L1 protein and the ATG5-ATG12-ATG16L1 complex, involved in the initiation of isolation membranes and targeting LC3 to autophagosomes, respectively, were subjected to further analysis. ATG9L1 was significantly decreased in SL (Figure 2a,d). Furthermore, while the above-mentioned proteins showed a granular layer-enriched localization, ATG5-ATG12 and ATG16L1 proteins were dominantly distributed in basal keratinocytes (Figure 2a). While there was no significant change in these targets, the signals were more extended from the basal to suprabasal layers in SL (Figure 2a,e,f).

**Figure 3:**
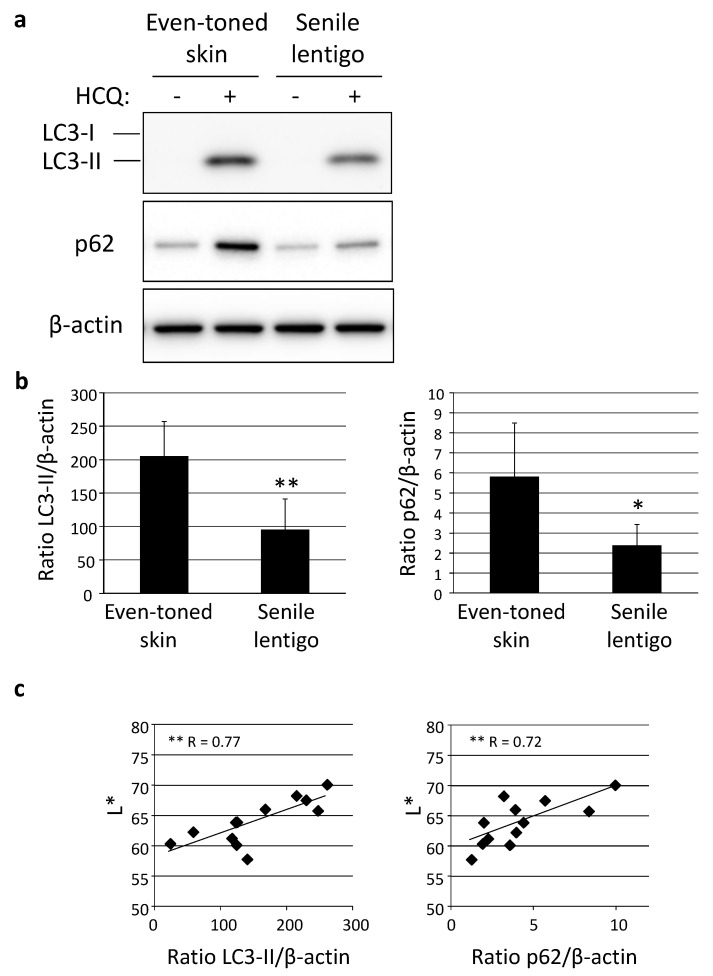
Decreased autophagy activity in senile lentigo. (**a**) Skin tissues obtained from even-toned and senile lentigo skin regions of Caucasian female subjects were cultured with or without 10 µM HCQ for 48 h, followed by western-blotting analysis using LC3- or p62-specific antibodies. β-actin = loading control. (**b**) After normalization with β-actin, the ratios of LC3-II (left panel) and p62 (right panel) under both conditions in the presence and absence of HCQ were compared. Values are shown as the mean ± SD from six independent subjects. ** *p* < 0.01; * *p* < 0.05 (paired t-test). (**c**) Correlations between ratios of LC3-II (left) and p62 (right), and chromameter-retrieved L* values. ** *p* < 0.01 (regression analysis).

Following immunohistochemical analysis, autophagic activity was examined in skin tissues using an autophagic flux assay, in which the turnover of both LC3-II and p62 can be estimated in the presence and absence of an autophagic inhibitor, such as hydroxychloroquine (HCQ) [17]. Western-blotting analysis clearly demonstrated a significantly lower level of autophagic activity in SL than in even-toned skin (Figure 3a,b). In addition, autophagy flux was shown to be significantly correlated with skin lightness (L*), determined by use of a chromameter (Figure 3c).

### 2.2. Autophagic Ability Significantly Depressed in Various Ranges of Hyperpigmented Skin Conditions

In order to globally comprehend the essential mechanism of autophagy in various skin phenotypes, a distinctive skin condition often observed in darker skin individuals was investigated. As compared to subjects with light- to medium-toned skin, predominant small pigmented spots, a skin discoloration typically observed in areas of sun-exposed joints (e.g., elbows and knees), is a skin problem often faced by darker skin individuals such as African Americans (AA). Both overall and close-up photographs clearly showed hyperpigmentation in the elbow regions of a representative AA subject (Figure S1a,b), with similar hyperpigmentation also observed on the knees (data not shown). In contrast, Caucasian subjects did not show this type of hyperpigmentation in these joint areas (Figure S1a,b). Notably, this type of hyperpigmented condition was often accompanied by a visibly dry and ashy skin appearance, which was even more apparent in non-polarized microscopic images and considered to be a distinct phenotype of the AA subjects (Figure S1b). Consistently, skin color was significantly different on elbows as compared with that on even-toned upper outer arm areas, in terms of skin lightness (L*), redness (a*), yellowness (b*), vividness (C*) and pigmentation (ITA°) (Figure S1c). In addition to skin color differences, elbow skin was also found to be significantly dehydrated, as shown by instrumental values obtained for skin capacitance and trans-epidermal water loss (TEWL) (Figure S1d). Skin capacitance and TEWL were both significantly correlated with skin color intensity, such as C* value (Figure S1e), which indicated that skin pigmentation and dehydration can coincidentally exist on dark joints.

**Figure 4:**
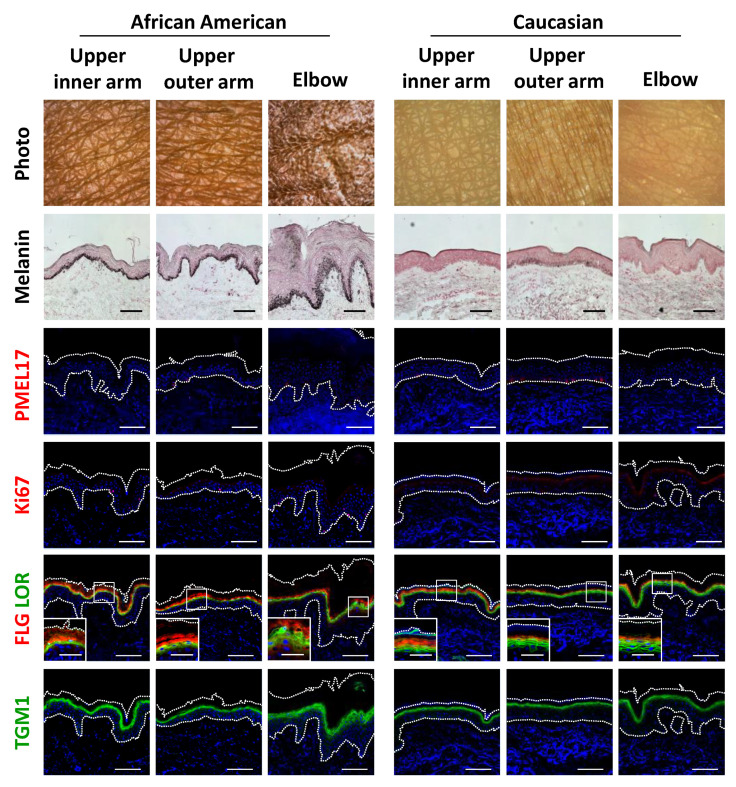
Disruption of epidermal differentiation leads to extensive melanin accumulation and abnormal epidermal development in dark joint regions. Biopsied skin samples were obtained from upper inner arm, upper outer arm and elbow areas of AA and Caucasian subjects, then subjected to Fontana-Masson staining and immunofluorescence observations. Merged images of PMEL17 (red), Ki67 (red), a set of filaggrin (FLG) (red) and loricrin (LOR) (green) or transglutaminase 1 (TGM1) (green), and DAPI nuclear staining are shown. Lower insets show magnification of areas inside white rectangles. Bars = 100 μm or 50 μm (insets).

Histological observations further demonstrated the unique features of darkly-pigmented and dehydrated elbow skin. Both the epidermis and stratum corneum layers were remarkably thickened, which was also accompanied by marked melanin deposition throughout the epidermis and stratum corneum (Figure 4). Surprisingly, immunofluorescent analysis showed that the abundance of PMEL17, a melanocytic marker protein, was not affected quantitatively or qualitatively in hyperpigmented elbow skin, whereas epidermal keratinocytes proliferation was found to be activated in the darkened elbow of an AA subject, as shown by measurements of Ki67 protein, a proliferation marker (Figure 4; Figure S2a,b). Furthermore, consistent with the histological results noted above, keratinocyte differentiation was disorganized in elbow skin, as indicated by irregular distributions and decreased amounts of the differentiation marker proteins filaggrin, loricrin and transglutaminase 1 (TGM1) in the AA subjects (Figure 4; Figure S2c–e,k). Furthermore, normally polarized localizations of filaggrin and loricrin were merged, and sometimes associated with swelling in hyperpigmented skin regions.

**Figure 5:**
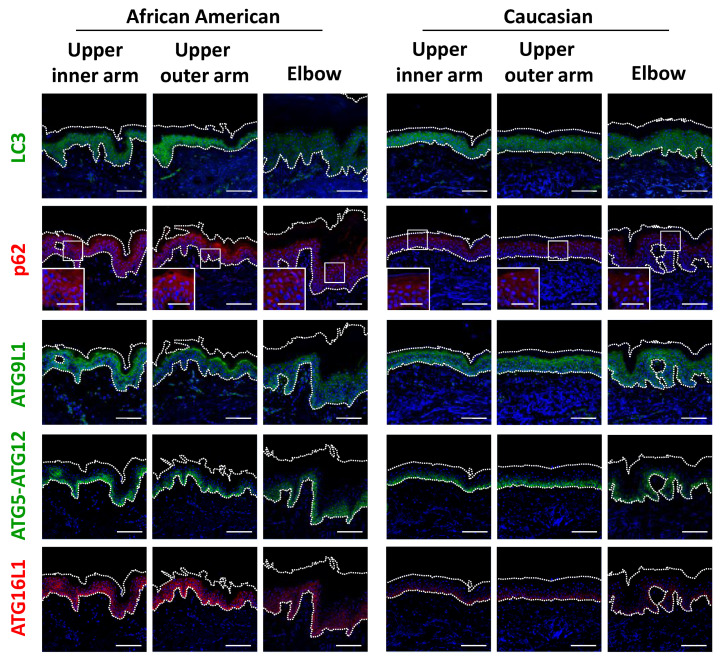
Impaired autophagy proteins in hyperpigmented skin. (a) Skin tissues from upper inner arm, upper outer arm and elbow regions of AA and Caucasian subjects were subjected to immunofluorescent analysis. Merged images of LC3 (green), p62 (red), ATG9L1 (green), ATG5-ATG12 (green) or ATG16L1 (red), and DAPI nuclear staining are presented. Lower insets show magnification of areas inside white rectangles. Bars = 100 μm or 50 μm (insets).

Based on the significant differences seen in regard to epidermal keratinocytes, we next focused on keratinocyte autophagy in the regulation of melanosome degradation, as well as epidermal differentiation. Consistent with findings showing accumulated melanin in the epidermis of elbow skin, the level of LC3 was remarkably decreased only in AA elbow skin (Figure 5 and Figure S2f). Accordingly, impaired autophagic activity was also reflected by p62 protein aggregation in AA elbow regions, as p62 is selectively degraded by the autophagy machinery (Figure 5). Additionally, other autophagy-related proteins, including ATG9L1, ATG5-ATG12 and ATG16L1, were significantly suppressed in the darker elbow regions of the AA subjects (Figure 5 and Figure S2h–j), despite the fact that these autophagic proteins were observed in similar samples obtained from Caucasian subjects.

**Figure 6:**
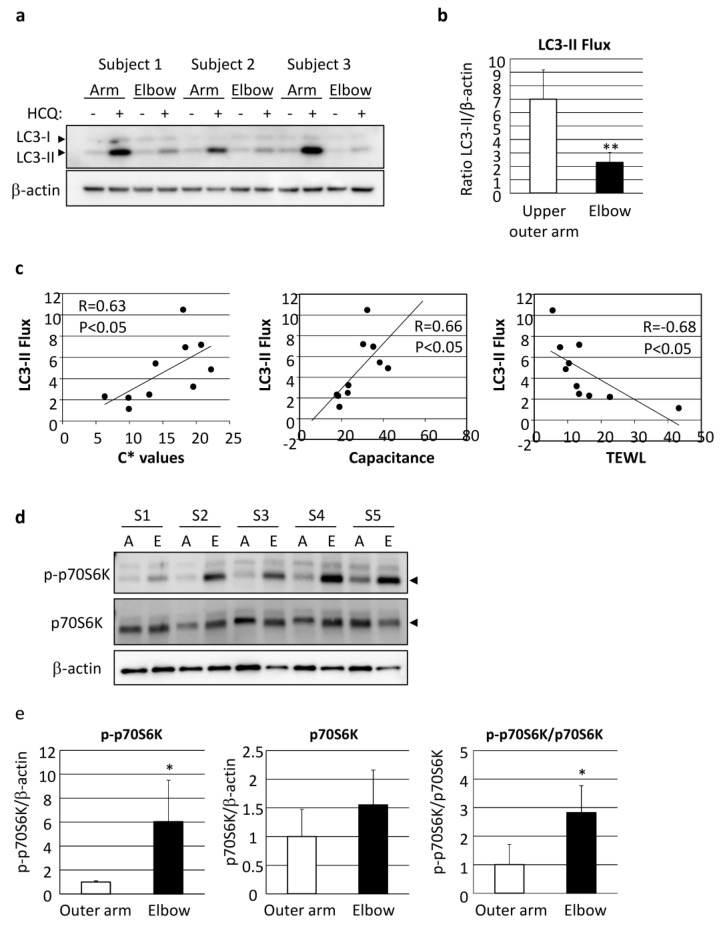
Depression of autophagic flux through stimulation of mTORC1 activity has significant impact on skin color and skin hydration. (**a**) Skin tissues obtained from upper outer arm (arm) and elbow regions of AA subjects were cultured with or without 10 µM HCQ for 48 h, followed by western-blotting analysis using an LC3-specific antibody. β-actin = loading control. (**b**) Following normalization with β-actin, the amount of LC3-II was compared between the presence and absence of HCQ. Values are shown as the mean ± SD from five independent AA subjects. ** *p* < 0.01 (paired t-test). (**c**) Correlations shown between LC3-II flux and C* (left), skin capacitance (center) and TEWL (right). Samples were obtained from the upper outer arm and elbow skin regions of five AA subjects. (**d**) Proteins were harvested from punch-biopsy-obtained skin tissues from the upper outer arm (A) and elbow (E) regions, and subjected to western blotting for phosphorylated-p70S6 kinase (p-p70S6K) or total p70S6K. β-actin = loading control. (**e**) Graphs show relative intensity of p-p70S6K (left), p70S6K (center) and p-p70S6K/p70S6K (right) after normalization. Values are shown as the mean ± SD (*n* = 5) and expressed as a ratio as compared with the upper outer arm region. * *p* < 0.05 (paired t-test).

Autophagy flux was also found to be significantly decreased in elbow skin as compared with even-toned skin on the upper outer arm of AA subjects (Figure 6a,b). It was interesting to note the correlation of LC3 flux with skin vividness (C* value), capacitance and TEWL, indicating that autophagy regulates both skin pigmentation and epidermal differentiation, including functional stratum corneum development (Figure 6c, Table 1).

We further attempted to determine the influence of the upstream factors involved in deteriorated autophagy. mTORC1 activity, as shown by phosphorylation of p70S6 kinase (p70S6K), was found to be significantly increased in the elbow skin of the AA subjects (Figure 6d,e).

### 2.3. Restoration of Autophagy by mTORC1 Inhibition Significantly Improved both Hyperpigmentation and Epidermal Differentiation in Tissue-Cultured Human Skin Ex Vivo

**Figure 7:**
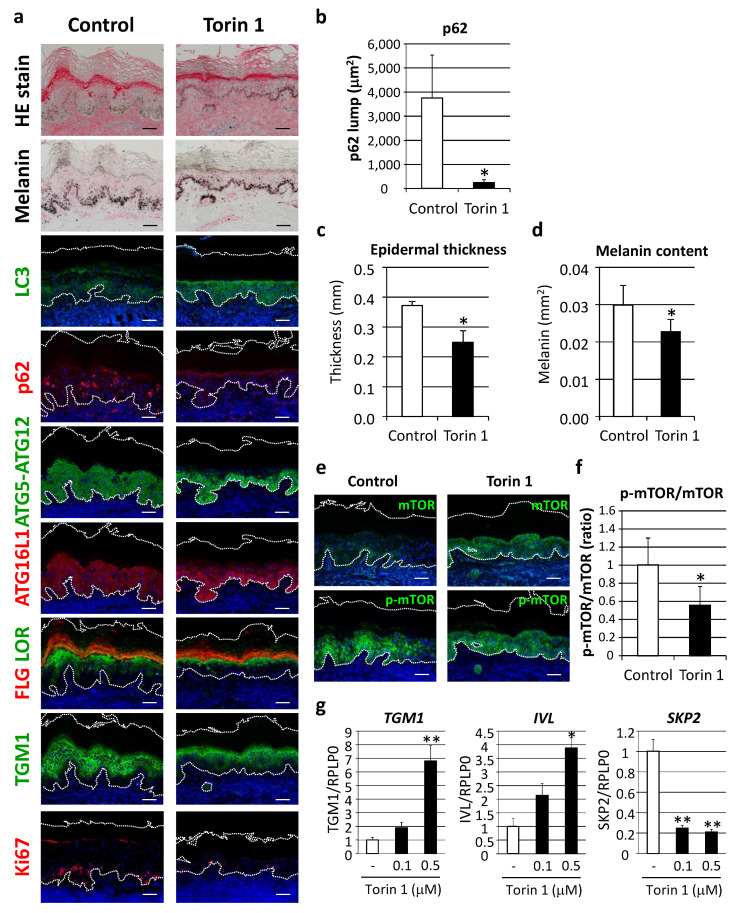
Inhibition of mTORC1 improved hyperpigmentation through restoration of autophagy. (**a**) Tissues obtained from AA elbow samples were treated with or without 1 μM Torin 1 for five days. Skin tissues cultured ex vivo were then subjected to hematoxylin and eosin (HE), and Fontana-Masson melanin staining for immunofluorescent analyses of LC3 (green), p62 (red), ATG5-ATG12 (green), ATG16L1 (red), FLG (red) and LOR (green) together, TGM1 (green), and Ki67 (red). Merged images with DAPI nuclear staining are shown. Bars = 100 μm. (**b**) Stained areas of aggregated p62 protein lumps were measured using the Image J software package. Values for the total areas of the p62 lumps are expressed as the mean ± SD (*n* = 4). * *p* < 0.05 (paired t-test). (**c**) Tissues with Fontana-Masson staining were subjected to Image J analysis to determine the thickness of the whole epidermis. Values are shown as the mean ± SD (*n* = 4). * *p* < 0.05 (paired t-test). (**d**) Relative areas positive for melanin per the length of stratum corneum were analyzed. Values show means ± SD (*n* = 4). * *p* < 0.05 (paired t-test). (**e**) Immunofluorescent analysis (green, top panels) and phosphorylation (green, bottom panels) of mTOR (p-mTOR) were performed after subjecting to nuclear staining with DAPI. Bars = 100 μm. (**f**) Fluorescent intensity was quantified and normalized based on the length of the basement membrane (BM). The ratio of p-mTOR and mTOR was compared between Torin 1-treated and -untreated tissues. Values are shown as the mean ± SD from 4 samples. * *p* < 0.05 (paired t-test). (**g**) Human normal epidermal keratinocytes (NHEKs) were treated with Torin 1 at the indicated doses for 24 h. mRNA transcript levels of *TGM1* (left), *IVL* (center) and *SKP2* (right) were determined using TaqMan real-time PCR, and their gene expression levels were normalized with those of *RPLP0* (ribosomal protein large P0). Values are shown as the mean ± SD from 3 samples. ** *p* < 0.01; * *p* < 0.05 (ANOVA, Dunnett’s test).

In order to verify the concept of autophagy restoration to improve the appearance of hyperpigmentation, as well as dysregulated epidermal differentiation, the effect of autophagy induction was evaluated in cultured human skin tissues ex vivo. Torin 1, a potent mTORC1 inhibitor, significantly restored LC3 protein along with decreased p62 protein aggregates (Figure 7a,b). Epidermal thickness and melanin deposition were confirmed to be significantly decreased by Torin 1 (Figure 7a,c,d) in parallel with normalized autophagic proteins, such as ATG5-ATG12 and ATG16L1 (Figure 5). In addition, keratinocyte differentiation and proliferation were ameliorated, as indicated by organized distributions of filaggrin, loricrin and TGM1 to the upper granular layer, along with suppression of Ki67-positive cells (Figure 7a). Torin 1-induced mTOR inhibition was also confirmed by findings of significant suppression of mTOR phosphorylation (Figure 7e,f).

In accordance with changes in cultured human skin tissues, treatment of normal epidermal keratinocytes with Torin 1 also led to an increase in epidermal differentiation proteins, such as TGM1 and involucrin, as well as a decrease in SKP2, a proliferation marker reported to inhibit autophagy via CARM1 [18] (Figure 7g).

## 3. Discussion

The present study examined autophagy in skin tissues to determine its physiological relevance to skin homeostasis, and the results showed decreased autophagy in regions of senile lentigo, a representative feature of skin discoloration associated with photoaging. Autophagy was also found to be deteriorated in areas with ashy hyperpigmentation, shown to be an extended damaged skin condition involving severe dehydration. Indeed, restoration by use of an autophagy activator ameliorated hyperpigmentation and epidermal tissue integrity ex vivo. Taken together, the present results show that the activation of autophagy is efficacious for regions with photoaging as well as other types of damaged skin via the concomitant regulation of pigmentation and epidermal function.

In association with photoaging, senile lentigo, characterized by brownish pigmented spots, often develops in skin that is frequently exposed to the sun. As for its mechanism, a continuous release of melanogenic cytokines from keratinocytes is considered to stimulate melanocyte activation [19,20]. Furthermore, evidence demonstrating an abnormal phenotype of keratinocyte differentiation has been presented. Some groups have independently reported decreased epidermal differentiation proteins in senile lentigo regions [15,16], suggesting an acquired change in regional keratinocytes, possibly caused by chronic sun exposure and aging. The present findings suggest that decreased autophagy plays a pleiotropic role in melanosome degradation, and potentially in keratinocyte differentiation, which will be discussed in the following. In a study that provided insight into further linkage between hyperpigmentation and aging, p53 was reported to be accumulated in pigmented spots in order to enhance melanogenesis [13]. Given that p53 can be activated by various types of cellular stress including aging, the accumulation of cellular damage caused by UV might further lead to premature aging, accompanied by a considerable increase in endophenotypes associated with autophagy inhibition. Collectively, the findings show that nuclear p53 mediates autophagy repression in a PINK1-dependent manner [21]. Decreased autophagy may also result in the accumulation of oxidative stress, as autophagy has been reported to remove oxidized phospholipids from keratinocytes [22]. Thus, autophagy induction might be a useful approach to combating this vicious cycle, as it was found to extend the lifespan of mice by suppressing oxidative stress and p53 [23].

Xerosis, a severe dry skin that can potentially occur at all ages, is thought to be a symptom of premature aging [24,25]. Chronic activation of p53 was reported to develop in an aging-associated xerosis phenotype by depleting sebaceous gland cells [26]. Although the present study did not investigate a broad range of xerosis conditions, including senile xeroderma, it was beneficial to examine areas of ashy skin, a unique skin condition possibly induced by various environmental stressors including UV and reduced humidity. Ashy hyperpigmentation is a common physiological condition that often develops in individuals with darker skin, and is characterized by a xerotic condition with a dull and dark skin tone [27,28]. Consistently, ashy hyperpigmentation showed a significant decrease in hydration status and apparent discoloration, as compared to healthy skin samples. Furthermore, darkly pigmented melanosomes were found to be distributed throughout the thickened epidermis from the basal layer to the stratum corneum, accompanied by parakeratosis. One of the important factors in this regard might be hyperactivation of the mTOR pathway, which promotes cell proliferation and survival. It is suggested that mTOR hyperactivation exacerbates the process of epidermal differentiation in hyperpigmented skin.

Regarding the role of autophagy and mTOR signaling in keratinocyte differentiation, there is an emerging body of evidence that has been obtained from investigations of skin diseases. Elevated phosphorylation of mTOR was reported in psoriatic skin, and was accompanied by the increased proliferation and disturbed differentiation of keratinocytes [29]. In addition, some autophagy proteins, such as LC3 and WIPI1, were found to be decreased in the lesional and non-lesional psoriatic epidermis, as compared with the healthy epidermis [4]. Accordingly, the present results clearly demonstrated that activation of mTOR, particularly mTORC1, may also be involved in physiological conditions such as xerotic ashy skin. It would also be of interest to investigate whether mTOR inhibition and autophagy induction are promising therapeutic options for skin disorders. Indeed, the mTORC1 pathway has been speculated as a druggable target for psoriasis [30]. As compared to the primary application of an mTOR inhibitor to diseased skin, it remains unclear how mTOR-dependent and -independent mechanisms maintain skin homeostasis through autophagy. Although the present study showed, at least in part, that mTOR inhibition directly leads to the differentiation of keratinocytes, an additional investigation is needed to unveil the role of autophagy in the whole process of epidermal cornification.

In addition, further interpretation of the immunohistochemical data regarding autophagy proteins is needed. Although the present results showed distributions of LC3 and ATG9L1 in granular layers, ATG5-ATG12 and ATG16L1 tended to be most abundant in basal and spinous keratinocytes, staining patterns in skin, which were findings consistent with previous reports [4,31]. One possible explanation is that mature autophagosomes labeled with LC3 are more enriched in differentiating keratinocytes. Although the ATG5-ATG12–ATG16L1 complex facilitates the modification of LC3 for its localization in autophagosomes [32], this complex is not associated with mature autophagosomes [33]. Indeed, the knockdown of LC3 was reported to lead to an increase in ATG5-ATG12- and ATG16L1-positive puncta [34]. Therefore, LC3 and perhaps other Atg8 orthologs, such as GATE-16/GABARAP, may be important effectors for autophagosome maturation in epidermal differentiation. Furthermore, decreased ATG9L1 may cause insufficient autophagy induction, as it is involved in the formation of isolation membranes and also facilitates LC3 lipidation under certain conditions [35]. Additional findings regarding transcriptional to post-translational regulation in autophagy machinery are needed.

Our results also provide a more comprehensive understanding of melanosome degradation in keratinocytes. Despite the predominant impact of the melanosomes in keratinocytes on skin pigmentation, little is known about the process involved or the scenario after melanosome transfer to keratinocytes. In agreement with our previous study [11], other groups have reported that lysosomes target melanosomes for degradation [36,37,38]. Further, Takano et al. noted a surprising effect of phenformin, a biguanide compound, on skin pigmentation by inhibiting melanosome degradation in keratinocytes [39]. Recently, Rab7B/42, a Rab family small GTPase, was revealed to target incorporated melanosomes for subsequent degradation in keratinocytes [40]. On the other hand, others have noted that internalized melanosomes are distributed within non-degradative intracellular compartments [41,42]. Hence controversy remains, and related studies should be conducted carefully with experimental conditions related to mono-layer cultures of keratinocytes, as there may be some limitations to uncovering the entire biological process related to melanosome degradation. Our results raise the possibility that melanosome degradation proceeds with keratinocyte differentiation in the epidermis, while a continuous re-organization of cellular proteins and organelles occurs in association with cornification. As Monteleon et al. recently noted, autophagic and lysosomal protein degradation is thought to be essential for the reorganization of proteins and organelles, while it also reduces total cellular biomass during keratinocyte differentiation [43]. With this perspective in mind, it is considered that melanosome degradation might also be governed in the same manner as that of other organelles, such as mitochondria and nucleus, in skin.

Another important aspect of the present investigation was the measurement of autophagy activity in human skin tissues to examine its contributions to skin condition. By integration of a conventional autophagy flux assay using human skin ex vivo cultures, we successfully determined autophagic activity in sampled skin tissues. Given recent findings regarding age-associated autophagy decline [3], further research would be warranted should autophagy be shown to also decrease with intrinsic skin aging, perhaps to varying degrees in different cell types, which could then lead to the consideration of an advanced approach to aging care by autophagy induction. Furthermore, diversity, including ethnic and individual differences, is an important factor to understand. For example, we recently identified a reciprocal interaction between autophagy and heat shock protein 70 (Hsp70) in generating ethnic variations in autophagic ability [44]. Hence, ethnicity or color-dependent variations in autophagy capacity in skin may be related to a susceptibility for damage-caused premature dysfunction, as highlighted by findings showing dehydrated hyperpigmentation in individuals with dark skin.

In conclusion, the present findings revealed that autophagy declines in association with premature human skin aging, including senile lentigo and ashy hyperpigmentation. Autophagy plays an essential role in maintaining multiple skin conditions, such as uniform skin color and skin barrier function, by controlling the integrity of melanosome degradation and epidermal differentiation.

## 4. Materials and Methods

### 4.1. Cell Cultures

Normal human epidermal keratinocytes (NHEKs) were purchased from Kurabo Co. (Osaka, Japan) and preliminarily incubated in EpiLife medium (Life Technologies, Carlsbad, CA, USA), as previously described [13].

### 4.2. Human Skin

Skin samples from senile lentigo and even-toned regions on the back were procured at Yaesu Nihonbashi Skin Clinic (Tokyo, Japan) from Japanese male subjects (42 to 57 years old) recruited by 701 Research Inc. (Tokyo, Japan). Collection of skin tissues was approved by the Institutional Review Board of Tokyo Sta. Center Building Clinic (Tokyo, Japan) (protocol numbers 12-053 and 14-0307). Punch-biopsy skin specimens, with or without SL, were obtained from the arms or shoulders of Caucasian females (44 to 56 years old) at RCTS, Inc. (Irvine, TX). Skin biopsy samples from the upper inner and outer arms, and the elbows of AA females (32 to 59 years old) and Caucasian females (33 to 50 years old) were also procured at RCTS, Inc. Collections of skin tissue samples performed in the US were approved by the Institutional Review Board of IntegReview Ltd. (Austin, TX, USA) (protocol numbers 2012.299, 2013.300, 2015.338). All sampling protocols were conducted according to the Declaration of Helsinki and written informed consent was obtained from each volunteer prior to the procedure.

### 4.3. Autophagy Flux Assay Ex Vivo

Skin biopsy tissues were cultured with or without 10 M HCQ for 48 h. Isolated proteins were subjected to western blotting analysis using anti-LC3 or -p62 antibodies to determine autophagic flux in the tissues [17].

### 4.4. Immunohistochemical Analysis

Biopsied skin samples were embedded in optical temperature cutting (OTC) compound. Frozen skin sections were fixed with cold acetone or 4% paraformaldehyde (PFA)/PBS. Tissues were then incubated in 1% bovine serum albumin (BSA)/PBS, followed by treatment with rabbit anti-LC3 antibody (Cell Signaling Technology, Danvers, MA, USA; 1:100), mouse anti-p62 antibody (MBL, Nagoya, Japan; 1:40), rabbit anti-ATG5 antibody (Sigma-Aldrich Co., St Louis, MO; 1:400), rabbit anti-ATG9L1 antibody (MBL; 1:400), goat anti-ATG16L1 antibody (LifeSpan BioSciences, Inc., Seattle, WA, USA; 1:300), mouse anti-Ki67 antibody (DAKO Inc., Carpinteria, CA, USA 1:100), mouse anti-filaggrin antibody (Abcam, Cambridge, UK; 1:500), rabbit anti-loricrin antibody (BioLegend, SanDiego, CA, USA; 1:1,000), rabbit anti-transglutaminase 1 antibody (Novus Biologicals, Littleton, CO; 1:400), mouse anti-PMEL17 antibody (DAKO; 1:40), rabbit anti-mTOR antibody or rabbit anti-phospho-mTOR antibody (Cell Signaling Technology, Danvers, MA, USA; 1:50 and 1:100). Incubation was performed with FITC-, RR-X- and/or Cy3-labeled secondary antibodies (Jackson ImmunoResearch Laboratories, Inc., West Grove, PA, USA; 1:100 or 1:200) corresponding to the primary antibody, followed by nuclear staining with 4′6-diamidino-2-phenylindole (DAPI) in a mounting solution (Vector, Burlingame, CA, USA). Images were obtained with a Zeiss LSM710 Confocal Microscope (Carl Zeiss Microscopy GmbH, Jena, Germany).

### 4.5. Fontana-Masson Staining

Tissue sections were fixed with 4% PFA and incubated in fontana ammonia silver solution. After washing, tissues were treated with kernechtrot solution, a nuclear fast red dye (Muto Pure Chemicals Co., Ltd., Tokyo, Japan), then mounted on Malinol slides (Muto). Images were obtained with an AxioVision microscope (Carl Zeiss). Areas positive for melanin relative to the total epidermal area were quantified using the Image J image analysis software package (Media Cybernetics, Rockville, MD), then statistically analyzed as previously reported [11,45].

### 4.6. Semi-Quantitative Image Analysis

The Image J software package was used for the quantification of immunofluorescent data. For RGB profile plots of immunofluorescence intensity, vertical distributions of fluorescent signals from the basal layer to stratum corneum were retrieved as a histogram using the RGB profile plot plug-in.

### 4.7. Western Blotting Analysis

Samples were solubilized in 0.1 mL RIPA buffer (Thermo Fisher Scientific, Rockford, IL) supplemented with a protease inhibitor cocktail (Roche, Rotkreuz, Switzerland) and homogenized using ultra-sonication. The resulting supernatants were collected as whole cell lysates and protein concentrations were determined using a BCA protein assay reagent (Pierce Biotechnology, Inc., Rockford, IL). After western blotting, membranes were incubated with diluted primary antibodies specific for LC3 (Cosmo Bio Co. Ltd., Tokyo, Japan or Life Technologies, Carlsbad, CA; 1:2,000), p62 (MBL International, Woburn, MA; 1:2000), phospho-p70S6K and p70S6K (Cell Signaling Technology, Danvers, MA; 1:1,000), or β-actin (Sigma-Aldrich Co.; 1:10,000). The blots were subsequently washed and incubated with diluted secondary antibodies (anti-mouse or -rabbit IgG peroxidase-linked F [ab’]2 fragment, GE Healthcare; 1:10,000). Immunoreactive bands were visualized with ECL Prime Western blotting detection reagents (GE Healthcare) and quantified using an ODYSSEY Fc Imaging system (LICOR Inc., Lincoln, NE). β-actin was used as an internal loading control standard.

### 4.8. Quantitative RT-PCR

Total RNA was extracted from NHEKs using an RNeasy Micro Kit (Qiagen, Valencia, CA, USA) then used for single-stranded cDNA synthesis with a High-Capacity cDNA Reverse Transcription Kit (Life Technologies). Quantitative Real-Time PCR (qPCR) was performed with a TaqMan Gene Expression Assay using a StepOnePlus™ Real-Time PCR System (Life Technologies). The specific probes and primers used for the target genes were *TGM1*, Hs00165929_m1; *IVL*, Hs00846307_s1; and *SKP*2, Hs01021864_m1 (Life Technologies). The expression of the genes of interest was normalized with that of *RPLP0* (ribosomal protein large P0, Hs99999902_m1) using a relative standard curve method for each target. The general conditions used followed the MIQE guidelines [46].

### 4.9. Non-invasive Skin Measurements

Microscopic photos were taken with an i-Scope^®^ (MORITEX Corporation, Saitama, Japan) at x50 magnification. Values for skin color were determined using a Minolta CR-300 Chromameter (KONICA MINOLTA, INC., Tokyo, Japan). Skin capacitance was read by a Corneometer^®^ CM 825 (Courage + Khazaka, Cologne, Germany). Trans-epidermal water loss (TEWL) was assessed with a TEWA meter 300^®^ (Courage + Khazakay).

### 4.10. Statistics

Statistical analyses of differences were calculated using Student’s t-test, a paired t-test, or ANOVA. *p* values <0.05 were considered to indicate a statistically significant difference. Significance of correlations was evaluated by regression analysis.

## Figures and Tables

**Table 1:** Correlations between autophagic activity and skin physiological properties.

Parameters	Correlation (R)
L*	0.41
A*	0.40
B*	0.68 *
C*	0.63 *
ITA	0.48
Capacitance	0.66 *
TEWL	−0.68 *

* *p* < 0.05 (regression analysis).

## Supplementary Materials

Explanation of the Supplementary Materials can be found at https://www.mdpi.com/1422-0067/21/16/5708/s1. Figure S1: Hyperpigmented ashy skin is a condition noted in African Americans. Figure S2: Semi-quantitative analysis following immunostaining of keratinocyte proliferation, differentiation and autophagy proteins.

## Abbreviations

AA	African American
ATG	autophagy-related protein
DAPI	4’,6-Diamidino-2-Phenylindole
HCQ	hydroxychloroquine
LC3	microtubule-associated protein light chain 3
mTOR	mammalian target of rapamycin
NHEK	normal human epidermal keratinocyte
PBS	phosphate-buffered saline
SL	senile lentigo
UV	ultraviolet
