# Supplementary material for: Autophagy Declines with Premature Skin Aging resulting in Dynamic Alterations in Skin Pigmentation and Epidermal Differentiation
Source: Int J Mol Sci. 2020 Aug 9;21(16):5708. doi: 10.3390/ijms21165708 (PMC7460956; doi:10.3390/ijms21165708)
Supplement: Supplementary file 1 [file ijms-21-05708-s001.zip › Murase et al. Supplemental figures.pdf]

Figure S1

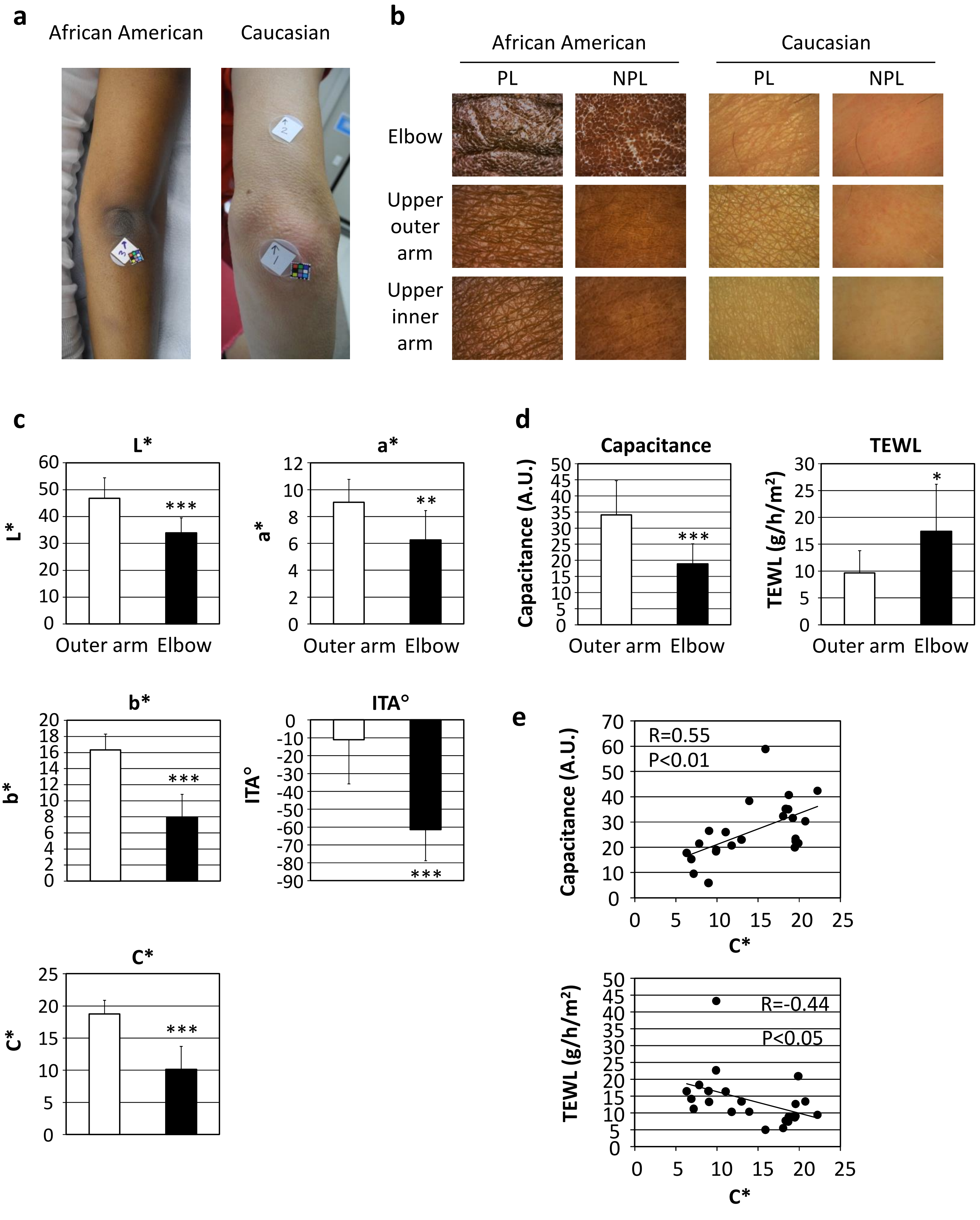
a
African American
Caucasian
b
African American
Caucasian
PL
NPL
PL
NPL
Elbow
Upper outer arm
Upper inner arm
c
L*
a*
b*
ITA°
C*
Outer arm
Elbow
***
**
d
Capacitance
Capacitance (A.U.)
TEWL
TEWL (g/h/m²)
Outer arm
Elbow
***
*
e
Capacitance (A.U.)
R=0.55
P<0.01
C*
TEWL (g/h/m²)
R=-0.44
P<0.05
C*

Figure S1. Hyperpigmented ashy skin is a condition noted in African Americans. (a) Photographs show clinical appearance of arms of female AA and Caucasian subjects. (b) Polarized (PL) and non-polarized (NPL) microscopic images of elbow, upper outer arm, and upper inner arm in female AA and Caucasian subjects. (c) Chromameter-retrieved skin color values are presented as L*, a*, b*, C*, and ITA°. Values are shown as the mean ± SD (n=12). ***P <0.0001; **P <0.01; *P <0.05 (paired-*t* test). (d) Skin hydration values from instrument measurements are shown. Capacitance (left panel) trans-epidermal water loss (TEWL) (right panel). ***P <0.0001; **P <0.01; *P <0.05 (paired-t test). (e) Correlations between chromameter-retrieved C* values and capacitance (top panel) or TEWL (bottom panel) are shown. **P <0.01; *P <0.05 (regression analysis).

Figure S2

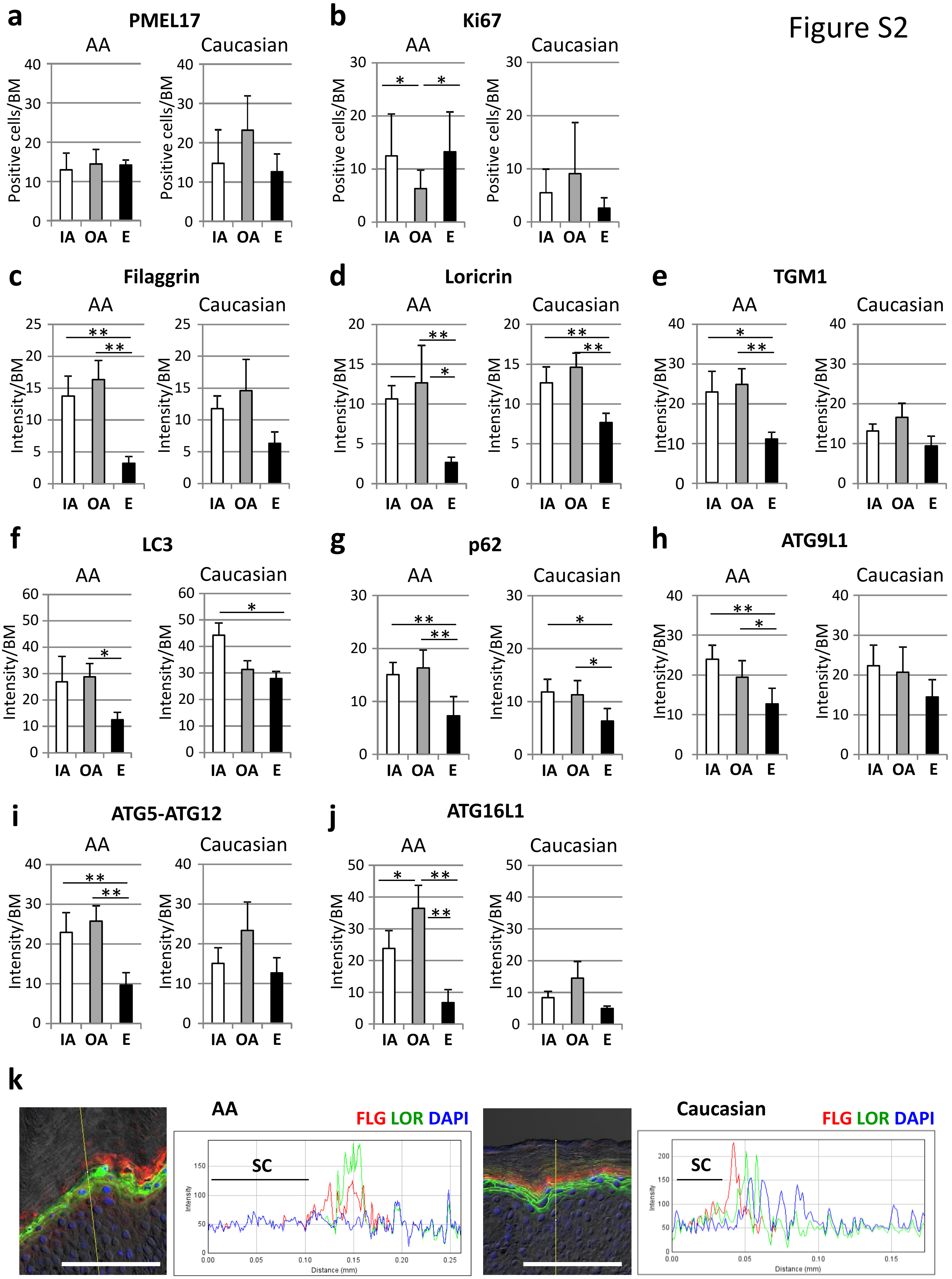

a PMEL17
AA
Caucasian
Positive cells/BM
IA OA E
b Ki67
AA
Caucasian
Positive cells/BM
IA OA E
c Filaggrin
d Loricrin
e TGM1
f LC3
g p62
h ATG9L1
i ATG5-ATG12
j ATG16L1
Intensity/BM
k
AA
Caucasian
FLG LOR DAPI
SC
Intensity
Distance (mm)

Figure S2. Semi-quantitative analysis following immunostaining of keratinocyte proliferation, differentiation, and autophagy proteins.

Immunohistochemical staining of Pmel17 and Ki67 proteins (Figure 2) was performed, then (a) Pmel17- and (b) Ki67-positive cells were counted, with their ratio to the length of the basement membrane (BM) shown. Values are shown as the mean ± SD (AA, n=4; Caucasian, n=3). **P <0.01; *P <0.05 (ANOVA, Bonferroni test). Results of immunofluorescence of (c) filaggrin, (d) loricrin, (e) TGM1, (f) LC3, (g) p62, (h) ATG9L1, (i) ATG5-ATG12, (j) ATG16L1 (shown in Figures 2 and 3) were subjected to analysis with Image J to determine the fluorescent intensity of each signal based on the length of the BM. Values are shown as the mean ± SD (AA, n=4; Caucasian, n=3). **P <0.01; *P <0.05 (ANOVA, Bonferroni test). (k) Fluorescent signal profiles following staining of FLG (red), LOR (green), and DAPI (blue) in elbow skin from AA (left) and Caucasian (right) subjects were analyzed using the RGB profile plot plug-in included with the Image J software package. Distributions of signal intensities were analyzed on the vertical axis (yellow line) and plotted from the stratum corneum (SC) to basement membrane. Bars = 100 μm.
